# Dynamic Evaluation of Notch Signaling-Mediated Angiogenesis in Ischemic Rats Using Magnetic Resonance Imaging

**DOI:** 10.1155/2018/8351053

**Published:** 2018-05-06

**Authors:** Jia-qi Tian, Jia-jun Zheng, Xiao-zhu Hao, Le-kang Yin, Xiao-xue Zhang, Chan-chan Li, Xiao-yuan Feng, Min Jiang, Hua-ping Sun, Kang Zheng, Yan-mei Yang

**Affiliations:** ^1^Department of Radiology, Huashan Hospital, Fudan University, Shanghai 200040, China; ^2^Department of Neurosurgery, Huashan Hospital, Fudan University, Shanghai 200040, China; ^3^Department of Radiology, Shanghai Chest hospital, Shanghai Jiao Tong University, Shanghai 200030, China; ^4^Department of Radiotherapy, Shanghai Eastern Hepatobiliary Surgery Hospital, Shanghai 310114, China; ^5^Institutes of Science and State Key Laboratory of Medical Neurobiology, Fudan University, Shanghai 200030, China

## Abstract

**Objective:**

The Notch signaling pathway is involved in angiogenesis induced by brain ischemia and can be efficiently inhibited by the *γ*-secretase inhibitor N-[N-(3,5-difluorophenacetyl)-1-alanyl]-S-phenylglycine t-butyl ester (DAPT). The aim of the present study was to noninvasively investigate the effect of DAPT treatment on angiogenesis in brain repair after stroke using magnetic resonance imaging (MRI).

**Methods:**

Sprague-Dawley rats (*n* = 40) were subjected to 90 minutes of transient middle cerebral artery (MCA) occlusion and treated with PBS (*n* = 20) or DAPT (*n* = 20) at 72 hours after the onset of ischemia. MRI measurements including T2-weighted imaging (T2WI), susceptibility-weighted imaging (SWI), and cerebral blood flow (CBF) were performed at 24 hours after reperfusion and weekly up to 4 weeks using a 3-Tesla system. Histological measurements were obtained at each time point after MRI scans.

**Results:**

SWI showed that DAPT treatment significantly enhanced angiogenesis in the ischemic boundary zone (IBZ) with respect to the control group, with local CBF in the angiogenic area elevated, along with increases in vascular density confirmed by histology.

**Conclusion:**

Treatment of ischemic stroke with DAPT significantly augments angiogenesis, which promotes poststroke brain remodeling by elevating CBF level, and these processes can be dynamically monitored and evaluated by MRI.

## 1. Introduction

Stroke is a major cause of mortality and disability globally while thrombolytic therapy as a fundamental treatment is not ideal for various restrictions and complications [1]. Obviously, there is a pressing need to develop effective strategies for the treatment of brain ischemia.

Neuroprotection as an alternative approach, which targets the cerebral parenchyma, is aimed at preserving cerebral tissue viability from reduced CBF. It seems to be a promising option for acute ischemic stroke (AIS) treatment. Unfortunately, most potentially neuroprotective drugs have failed to show benefit in clinical trials. At present, there is an increasing amount of studies that focus on the endogenous brain repair processes after stroke, which constitute the basis of neurorestorative therapy. As an essential part of biological substrates for poststroke brain remodeling, angiogenesis induced by brain ischemia enhances blood flow and nutrient supply to the affected tissue, which may benefit functional recovery [2, 3]. In rodents, neurorestorative treatments of either cell-based or pharmacological therapies promote angiogenesis, which is associated with improvements in functional outcome [4, 5]. Patients with higher density of blood vessels seem to survive longer than patients with lower vascular density [6]. Moreover, angiogenesis and neurogenesis might be closely and causally linked together [7]. Therefore, the modulation of the endogenous angiogenesis could be a potential therapeutic intervention that promotes functional recovery after stroke.

The Notch signaling pathway plays multiple roles during vascular development, physiology, and disease [8]. Increasing evidence has pointed out that this pathway appears to regulate several steps of the reparative process occurring in ischemic tissues [9, 10]. However, the association between Notch signaling-mediated angiogenesis and poststroke brain remodeling via the improved CBF has not been investigated.

Magnetic resonance imaging (MRI) acts as a useful tool to noninvasively assess the evolution of various indices that can characterize postischemic angiogenic processes, which have been demonstrated from a variety of studies in experimental stroke models [11–13]. The aim of this study was to dynamically evaluate the temporal and spatial changes of angiogenesis modulated by Notch signaling in cerebral ischemia with noninvasive MRI methods.

## 2. Materials and Methods

All experimental procedures were performed in accordance with the National Institutes of Health Guide for the Care and Use of Laboratory Animals and approved by the Institutional Animal Care and Use Committee of Fudan University. Every effort was made to minimize the number of animals used and their suffering.

### 2.1. Animal Model and Experimental Design

Adult male Sprague-Dawley rats (*n* = 40) weighing 260–270 g were subjected to 90 minutes of transient middle cerebral artery occlusion (tMCAO) [14]. Briefly, rats were anesthetized with 10% chloral hydrate (350–400 mg/kg) intraperitoneally. The body temperature of 37 ± 0.5°C was kept. The left MCA was occluded with a 4.0 silicon-coated nylon filament (Beijing Cinontech Biotech Co. Ltd., Beijing, China). It was inserted into the internal carotid artery (ICA) through the left common carotid artery (CCA) to occlude the origin of the MCA. After 90 minutes, the animals were reperfused by removing the filament.

We randomly divided the rats into control (*n* = 20) and treated groups (*n* = 20). DAPT (Sigma-Aldrich, St. Louis, MO, USA) powder was dissolved in DMSO at the concentrations of 8.3 mg/ml and stereotactically injected into the lateral cerebral ventricle (LV) for the treated group rats (0.03 mg/kg) at 3 days after stroke [15]. The detailed experimental procedure had been previously described [16]. The same volume of PBS was given to the control group rats at 3 days.

**Figure 1:**
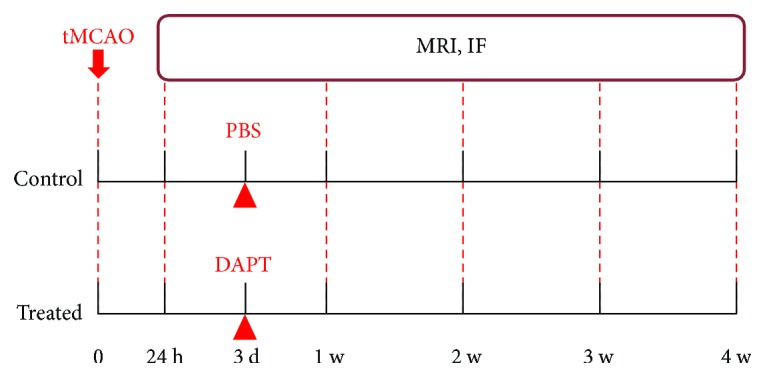
Experimental design. Forty rats were subjected to 90 minutes of transient middle cerebral artery occlusion (tMCAO) and then treated with PBS or DAPT at 3 days after ischemia. MRI was performed at 24 hours and weekly up to 4 weeks, after which four rats of each group were decapitated, respectively, for IF.

Four rats of each group were decapitated at 1 day and weekly up to 4 weeks after MRI scan, respectively, for immunofluorescence staining (IF).  Figure 1 shows the time schedule for the experimental design.

### 2.2. Magnetic Resonance Imaging Measurements

All rats received MR scanning using a 3-Tesla MR system (Discovery MR750; GE Medical Systems, Milwaukee, WI, USA) with a 60 mm diameter gradient coil (Magtron Inc., Jiangyin, China). The total acquisition time was 18 minutes. Anesthesia was maintained during the imaging procedure as mentioned above. Body temperature was kept at 37 ± 0.5°C. Blood oxygen saturation and heart rate were monitored.

MR images including T2-weighted imaging (T2WI), susceptibility-weighted imaging (SWI), and three-dimensional arterial spin labeling (3D-ASL) were acquired for all animals in both groups at 1 day and weekly up to 4 weeks after the onset of stroke.

T2WI images were scanned using a fast spin-echo sequence by multiple slices (15 slices; 1.8 mm slice thickness and 1 mm interslice distance). Repetition time (TR) and echo time (TE) were 4000 ms and 96 ms, respectively. Images were produced using a 6 × 6 cm FOV and a 256 × 256 matrix. The scan time was 3 min.

SWI employed a 3D gradient echo imaging sequence by multiple slices (30 slices; 1 mm slice thickness and 1 mm interslice distance). RT and ET were 56 ms and 32 ms, respectively. The matrix was set as 300 × 300 for the 7 × 7 cm FOV. The flip angle is 10°. The sequence time was approximately 10 minutes.

3D-ASL was acquired by a 3D spiral fast spin-echo sequence with the following parameters: repetition time (RT) = 4214 ms (PLD = 1.5 s) and 5285 ms (PLD = 2.5 s), labeling duration = 1500 ms, echo time (ET) = 11.948 ms, scan time = 4 min, field of view (FOV) = 4 × 4 cm, slice thickness (ST) = 3 mm, and interslice distance = 3 mm.

### 2.3. MRI Data Analysis

**Figure 2:**
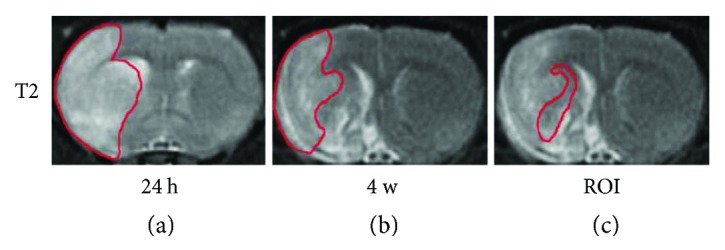
Representative T2 maps indicating the ROI. (a) Ischemic lesion size was identified by the T2 map acquired at 24 h after stroke. (b) The final infarction area was determined by the T2 map acquired at 4 weeks. (c) The difference was referred to as the recovery region, the ROI.

The MR images were postprocessed using the Functool software based on GE Advanced Workstation 4.6 (GE Medical Systems, Milwaukee, WI). Ischemic lesion size was determined by T2 maps acquired after stroke with values above mean plus two standard deviations (SD) of contralateral measurements [11]. The region of interest (ROI), identified by the difference of the ischemic lesion sizes in T2 maps acquired at 24 h (Figure 2(a)) and 4 weeks (Figure 2(b)) after stroke, was referred to as the recovery region (Figure 2(c)). This region and the mirrored area in the contralateral hemisphere were chosen to measure MRI values, and ratios were obtained. All the ROI were animal dependent.

### 2.4. Histology

After MRI measurements at each time point, rats were euthanized and transcardially perfused with 200 ml of 0.9% saline followed by 200 ml of 4% paraformaldehyde. The brains were removed and postfixed in 4% paraformaldehyde at 4°C overnight. Coronal brain sections (20 *μ*m) were obtained using a cryostat (RM2135; Leica, Mannheim, Germany) and stained for histological evaluation. The detailed procedure had been previously described [16]. Primary antibodies of rabbit polyclonal anti-NICD (1 : 500; Abcam, Cambridge, MA, USA) and mouse monoclonal anti-CD31 (1 : 100; Abcam, Cambridge, MA, USA) were used to evaluate the cerebral vascular density and Notch signal inhibition. Alexa Fluor 488- and 568-conjugated donkey anti-rabbit and anti-mouse (1 : 200; Life Technologies, Carlsbad, CA, USA) were used as secondary antibodies. Sections were then counterstained with DAPI (1 : 1000; Sigma-Aldrich, St. Louis, MO, USA).

Histological images were scanned using a fluorescence microscope (Olympus PX51; Olympus Corporation, Shinjuku-ku, Japan). The acquired images were quantified using ImageJ (National Institutes of Health, Bethesda, MD, USA) by measuring optical density of positively stained cells of the ROI selected at the recovery region. Five sections of each brain from each group were used for cell counting at 40x objective in three randomly selected views per section. The ipsilateral-versus-contralateral differences in gray levels were converted to a percentage of the contralateral value.

### 2.5. Statistical Analysis

All values were presented as mean ± SD. The temporal patterns of MRI data between two groups, including CBF and SWI, were analyzed using two-way analysis of variance (ANOVA) with Sidak's multiple comparison test. The differences of histological measurements between the 2 groups were analyzed by *t*-test. A value of *P* < 0.05 was considered statistically significant. Statistical analysis was performed using Prism, version 6.0 (GraphPad Software Incorporated, La Jolla, CA, USA).

## 3. Results

### 3.1. MRI Measurements

**Figure 3:**
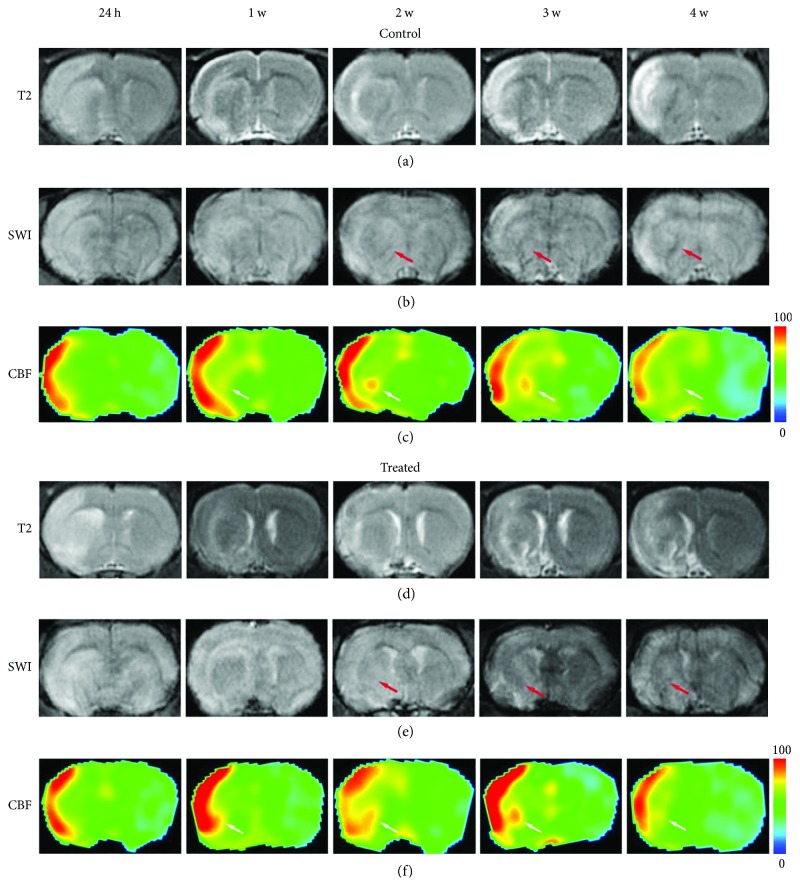
MRI maps of representative control (a–c) and treated (d–f) rats. (c, f) Elevated CBF (white arrows) was found in the recovery region. (b, e) SWI showed hypointensity in the same area (red arrows) indicating angiogenesis. The area where angiogenesis might happen in the control rat was smaller than that in the treated one.

Figure 3 shows the evolution of an axial section of MRI maps from the representative control rat (a–c) and treated rat (d–f). Ischemic lesion and temporal evolution of the two groups after ischemia were identified by T2 images (Figures 3(a) and 3(d)). Elevated CBF was observed in cortical regions since 24 h in both groups (Figures 3(c) and 3(f)). Elevation of CBF values had appeared at 1 week in striatum regions in the control rats and treated rats, as indicated by white arrows. These areas were detected as having low intensity on the SWI map (Figures 3(b) and 3(e)), which had appeared at 2 weeks in the two groups, as indicated by red arrows. For the treated rats, the size of the hyperintensity areas on the CBF map and hypointensity regions on the SWI map was larger than that in the control rats.

**Figure 4:**
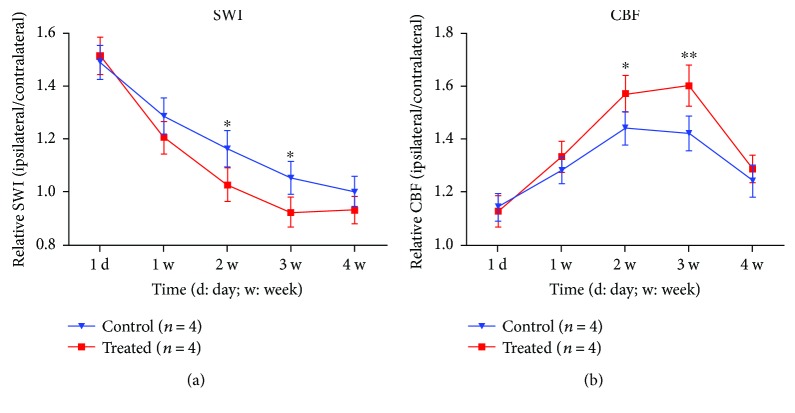
Quantitative MRI data demonstrating temporal features of SWI and CBF for both groups. (a) SWI ratios of cerebral tissue in the DAPT-treated group had lower values during 4 weeks after stroke compared to those in the control group; the differences were significant at 2 weeks and 3 weeks. (b) Higher CBF ratios were observed in the DAPT-treated group compared to control rats during 4 weeks; the differences were significant at 2 weeks and 3 weeks. ^∗^*P* < 0.05; ^∗∗^*P* < 0.01.

Quantitative longitudinal MRI measurements demonstrated temporal features of CBF and SWI for restorative cerebral tissue after stroke with or without DAPT treatment (Figure 4). As shown in Figure 4(b), elevated CBF ratios had a good consistency between the two groups at 24 h following ischemia. For the control rats, CBF increased starting from 1 to 2 weeks and then regressed toward normal from 3 weeks. For the treated rats, CBF increased starting from 1 to 3 weeks and then decreased at 4 weeks. Compared to the control rats, CBF of cerebral tissue in IBZ in the treated group had consistently higher values during 1 to 4 weeks after stroke. The differences were significant at 2 and 3 weeks (*P* < 0.05 and 0.01, resp.).

Temporal changes of SWI ratios for both treated and control groups were shown in Figure 4(a). It demonstrated a good consistency between the two groups at 24 h. SWI ratios monotonically decreased during 4 weeks after stroke for the control group. For the treated rats, SWI ratios decreased starting from 1 week and reached to minimum at 3 weeks and then elevated at 4 weeks. Compared to the control rats, SWI ratios in the treated group had consistently lower values during 1 to 4 weeks. The differences were significant at 2 and 3 weeks (*P* < 0.05).

### 3.2. Histological Measurements

**Figure 5:**
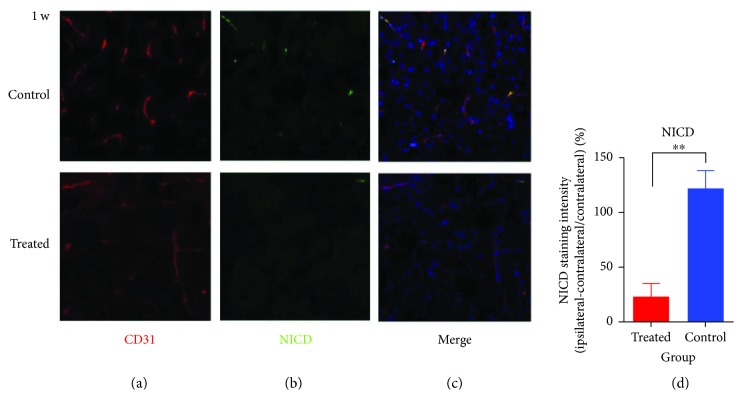
Expression of CD31 and NICD indicating the Notch signal blocked by DAPT. (a–c) Immunostaining of NICD-positive microvessels in IBZ for both groups obtained 1 week after stroke (40x). (d) Quantification of NICD fluorescence intensity. ^∗∗^*P* < 0.01.

Double immunofluorescent staining (Figures 5(a)–5(c)) showed the NICD-positive microvessels in IBZ in both groups at 1 week (40x microscope). Quantitative analysis of NICD-positive microvessels showed that the number was higher in the control group than in the treated group (Figure 5(d), *P* < 0.01), which indicated that the Notch signal was blocked by DAPT.

**Figure 6:**
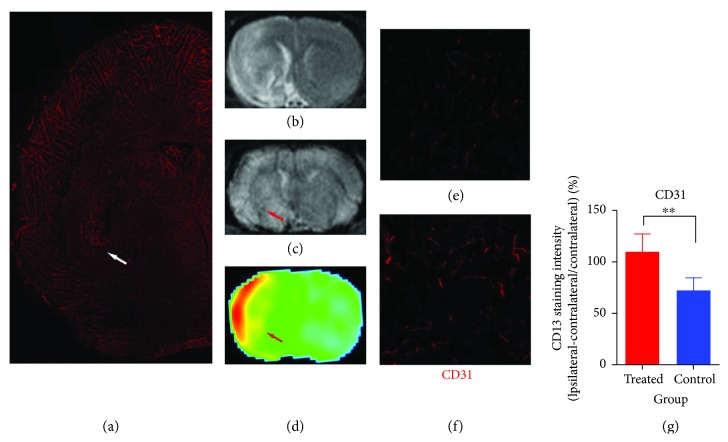
Histology and MRI matched the area indicating angiogenesis. (a) A CD31-stained slice and (b–d) MR images of a treated rat obtained at 4 weeks after stroke. These areas are close matched (arrows). (e, f) Immunostaining of CD31-positive cerebral vessels in IBZ for both groups obtained 4 weeks after stroke (20x). (g) Quantification of CD31 fluorescence intensity. ^∗∗^*P* < 0.01.

MRI data for detecting angiogenesis in this study were confirmed by histological measurements using CD31 staining, as shown in Figure 6. A CD31-stained slice (Figure 6(a)) of a treated rat was obtained at 4 weeks after stroke. The T2 image (Figure 6(b)), SWI map (Figure 6(c)), and CBF map (Figure 6(d)) obtained 4 weeks after stroke detected the angiogenic area in the IBZ (red arrows). This area closely matched the histological result (white arrow).

Figures 6(e) and 6(f) show the representative pictures (20x microscope) of CD31-immunoreactive cerebral vessels for ipsilateral slices of control and DAPT-treated rats in the IBZ area obtained 4 weeks after stroke, respectively. Compared to the control rats, treatment with DAPT significantly increased cerebral vessel density (Figure 6(g), *P* < 0.01).

## 4. Discussion

Our study showed that the number of NICD-positive microvessels in the control group was higher than that in the treated group at 1 week, which indicated that the Notch signal was blocked by DAPT at 3 days. Cerebral vessel density was significantly enhanced in treated rats at 4 weeks after stroke compared to the control rats, suggesting that inhibition of the Notch signal significantly promoted angiogenesis. It is now becoming clear that the Notch signaling pathway plays a significant role in both developmental and pathological angiogenesis [9, 17]. Ischemia triggers endogenous angiogenesis, involving the new capillaries sprouting from previously existing blood vessels. The current model of endothelial angiogenesis centers on the interplay between “tip” and “stalk” cell characters, and Notch signaling acts as a key regulator of endothelial tip cell formation and function during this process [10, 18]. Suppression of Notch signaling leads to increased endothelial cell proliferation with augmented sprouting and excessive vascular branching [19, 20]. Our study is consistent with previous studies that angiogenesis after ischemia was enhanced by blocking Notch signaling. Together, Notch signaling seems to be essential in building up a proper neovascularization, and the detailed process of how exactly Notch signaling regulates angiogenesis requires further investigations.

Experimental studies suggest that cell-based and pharmacological neurorestorative treatments can promote brain plasticity and benefit functional recovery [2, 3]. However, the current understanding of these processes after stroke derives mainly from regional histological measurements, which do not allow dynamic assessment of tissue remodeling [21]. The high spatial and temporal resolution, safety, and versatility make MRI particularly suitable to monitor the dynamic profiles of events involved in poststroke brain reorganization. After proliferation of endothelial cells, newly formed vessels in the ischemic border zone lead to alterations in vascular density and hemodynamic parameters such as cerebral blood flow (CBF), which can be measured with perfusion MR imaging techniques such as arterial spin labeling (ASL) [22]. MRI-based measurements of elevated CBF were evident in ischemic boundary regions with neural progenitor cell-induced angiogenesis in an embolic stroke model [12]. Similarly, Ding et al. [11] and Li et al. [23] have reported that treatment with sildenafil enhanced angiogenesis and selectively increased the CBF level in the ischemic boundary in rats after embolic stroke. Newly generated venous structures can be identified with susceptibility-weighted images. The local magnetic field disturbances caused by the relatively high magnetic susceptibility of deoxygenated hemoglobin result in a lower signal intensity on the SWI map, which has been used to detect perilesional angiogenesis in sildenafil- or erythropoietin-treated rats after ischemia [13, 24]. Our dynamic measurements revealed that elevation of CBF values appeared at 1 week in striatum regions in the two groups and the closely matched area with low intensity on the SWI map appeared at 2 weeks in the two groups. The size of the hyperintensity areas on the CBF map and hypointensity regions on the SWI map was larger in treated rats compared to the control group. CBF of cerebral tissue in IBZ in the treated group had consistently higher values during 1 to 4 weeks after stroke while SWI ratios had lower values during 1 to 4 weeks. These results suggest that poststroke angiogenesis is augmented by blocking Notch signaling, and this process can be evaluated by noninvasive MRI methods.

Compensatory angiogenesis after ischemic brain injury is considered an intrinsic process of the brain for neuroplasticity. It is essential for poststroke brain repair, as this event stimulates blood flow and nutrient supply to the affected tissue [25]. In stroke patients, the extent of angiogenesis is associated with survival and improved neurological recovery [6]. In animal stroke models, neurorestorative treatments of either cell-based or pharmacological therapies promoted angiogenesis, which is associated with improvements in functional outcome [4, 5]. Our previous study showed that suppression of Notch signaling resulted in ameliorated brain edema at the subacute stage and recovered brain tissue at the chronic stage in the ipsilateral striatum, as indicated by MRI data [16]. Evidently, this is partially due to the ischemia-induced angiogenesis. In addition, the benefits probably come through the interplay between neurorestorative events (angiogenesis, neurogenesis, and synaptic plasticity), which will be investigated in our future experiment.

## 5. Conclusion

The current study demonstrates that treatment of ischemic stroke with DAPT by suppression of the Notch signal significantly augments angiogenesis, which promotes poststroke brain remodeling by elevating CBF level. Noninvasive MRI methods can be used to investigate and evaluate cerebral tissue undergoing angiogenesis and can be helpful in estimating the potential in functional recovery, all of which may provide new insight into clinical trials of angiogenic therapy for cerebral ischemia.
